# Targeted delivery of anti-miRNA21 sensitizes PD-L1^high^ tumor to immunotherapy by promoting immunogenic cell death

**DOI:** 10.7150/thno.97755

**Published:** 2024-06-17

**Authors:** Eun Hye Kim, Jiwoong Choi, Hochung Jang, Yelee Kim, Jong Won Lee, Youngri Ryu, Jiwon Choi, Yeonho Choi, Sung-Gil Chi, Ick Chan Kwon, Yoosoo Yang, Sun Hwa Kim

**Affiliations:** 1 Medicinal Materials Research Center, Biomedical Research Division, Korea Institute of Science and Technology (KIST), Seoul 02792, Republic of Korea.; 2 Department of Life Sciences, Korea University, Seoul 02841, Republic of Korea.; 3 Division of Bio-Medical Science and Technology, KIST School, University of Science and Technology, Seoul 02792, Republic of Korea.; 4 KU-KIST Graduate School of Converging Science and Technology, Korea University, Seoul 02841, Republic of Korea.; 5 Department of Bioengineering, Korea University, Seoul 02841, Republic of Korea.

**Keywords:** Anti-miRNA delivery, PD-L1, miR-21, Tumor microenvironment, Immunogenic cell death.

## Abstract

**Rationale:** Growing evidence has demonstrated that miRNA-21 (miR-21) upregulation is closely associated with tumor pathogenesis. However, the mechanisms by which miR-21 inhibition modulates the immunosuppressive tumor microenvironment (TME) and improves tumor sensitivity to immune checkpoint blockade therapies remain largely unexplored. In this study, we demonstrate the precise delivery of anti-miR-21 using a PD-L1-targeting peptide conjugate (P21) to the PD-L1^high^ TME.

**Methods:** Investigating miR-21 inhibition mechanisms involved conducting quantitative real-time PCR, western blot, flow cytometry, and confocal microscopy analyses. The antitumor efficacy and immune profile of P21 monotherapy, or combined with anti-PD-L1 immune checkpoint inhibitors, were assessed in mouse models bearing CT26.CL25 tumors and 4T1 breast cancer.

**Results** Inhibition of oncogenic miR-21 in cancer cells by P21 efficiently activates tumor suppressor genes, inducing autophagy and endoplasmic reticulum stress. Subsequent cell-death-associated immune activation (immunogenic cell death) is initiated via the release of damage-associated molecular patterns. The *in vivo* results also illustrated that the immunogenic cell death triggered by P21 could effectively sensitize the immunosuppressive TME. That is, P21 enhances CD8^+^ T cell infiltration in tumor tissues by conferring immunogenicity to dying cancer cells and promoting dendritic cell maturation. Meanwhile, combining P21 with an anti-PD-L1 immune checkpoint inhibitor elicits a highly potent antitumor effect in a CT26.CL25 tumor-bearing mouse model and 4T1 metastatic tumor model.

**Conclusions:** Collectively, we have clarified a miR-21-related immunogenic cell death mechanism through the precise delivery of anti-miR-21 to the PD-L1^high^ TME. These findings highlight the potential of miR-21 as a target for immunotherapeutic interventions.

## Introduction

MicroRNAs (miRNAs), a class of endogenous small non-coding RNA, epigenetically regulate gene expression 1, 2. However, aberrant miRNA expression is often associated with cancer, leading to dysregulated gene expression that controls tumor biology 3, 4. In particular, the ubiquitous miRNA-21 (miR-21) is the most commonly upregulated cancer-associated miRNA and is closely linked to poor prognosis 5, 6. The role of miR-21 in tumorigenesis has been extensively studied in cancer cells, revealing its regulatory roles in various downstream signaling pathways associated with cell proliferation 7, migration 8, 9, apoptosis 10, and chemoresistance via suppression of pro-apoptotic protein expression, including phosphatase and tensin homolog (PTEN) and programmed cell death protein 4 (PDCD4).

In addition to cancer cells, miR-21 is highly expressed in various hematopoietic cells of the immune system, including dendritic cells (DCs), macrophages, T lymphocytes, and B lymphocytes 11, 12. Recent studies have highlighted the role of miR-21 as a negative regulator in pro-inflammatory responses; for instance, heightened miR-21 levels in bone marrow-derived DCs (BMDCs) have been linked to the inhibition of DC maturation 13, 14. Nevertheless, the functions of miR-21, which are contingent on cellular and microenvironmental factors, remain insufficiently characterized. Moreover, the mechanisms by which miR-21 inhibition modulates the immunosuppressive tumor microenvironment (TME) remain controversial 15-17. While miR-21 manipulation has been shown to elicit notable antitumor effects *in vivo*, findings relying on *in vitro* culture systems that inhibit or overexpress miR-21 have led to inconsistent conclusions 18. Indeed, the induction of tumor cell apoptosis following miR-21 downregulation cannot entirely account for its tumor-suppressive effect *in vivo*. Additionally, recent studies aimed at deciphering the role of miR-21 within the TME have yielded conflicting results 19-21, often attributed to the lack of cell-type-specific targeting approaches. Hence, the clinical application of targeting miR-21 as an anticancer therapy requires further investigations addressing the underlying mechanisms of miR-21 inhibition via target-specific delivery systems within the TME.

**Scheme 1:**
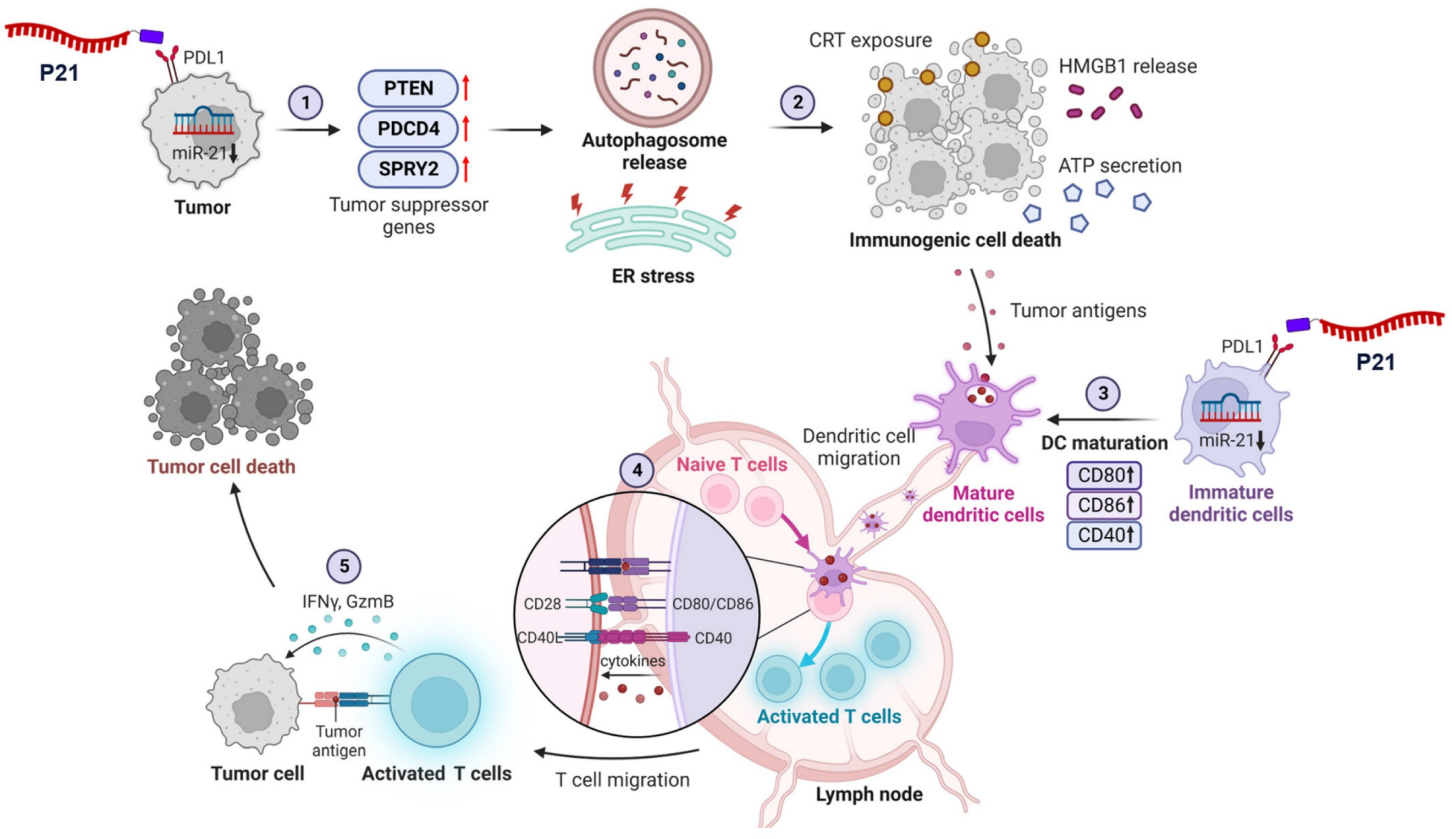
** Schematic of P21-mediated cancer immunotherapy.** 1) Downregulation of oncogenic miR-21 by P21 in cancer cells can activate tumor suppressor genes. 2) PTEN/PDCD4-mediated ICD induces ER stress and autophagy. 3) P21-induced DAMP exposure activates DC functions. 4) Promoting DC maturation increases CD8^+^ T cell infiltration in tumor tissues. 5) P21 effectively enhances antitumor immunity through CD8^+^ T cell activation.

In this study, we investigate the mechanisms by which inhibiting miR-21 expression reverses the immunosuppressive TME and triggers antitumor immune responses **(Scheme 1)**. To this end, we used previously developed peptide-oligonucleotide conjugate targeting PD-L1^high^ tumor cells 22, in which an inhibitor of oncogenic miR-21 (anti-miR-21) was directly bound to a PD-L1-binding peptide (P21). For the first time, we uncover that inhibition of oncogenic miR-21 by P21 can activate signaling pathways associated with PTEN/PDCD4-mediated immunogenic cell death (ICD) by inducing endoplasmic reticulum (ER) stress and autophagy. Our results also show that decreasing miR-21 expression in BMDCs promotes DC maturation, leading to CD8^+^ T cell-dependent immune responses. Importantly, inhibiting miR-21 in the TME increases anti-PD-L1 antibody therapy susceptibility, reducing primary tumor growth and lung metastasis.

Recently, there has been growing interest in understanding the role of miRNAs as pivotal regulators in orchestrating anticancer immune responses to control tumor development. Consequently, the TME-specific targeting of miR-21 for ICD regulation holds considerable potential for enhancing the clinical relevance of miRNA therapeutics. Accordingly, this approach represents a promising synergistic strategy for cancer immunotherapy.

## Results

### Inhibition of miR-21 triggers ICD via activation of tumor suppressor genes in PD-L1^high^ cancer cells

**Figure 1:**
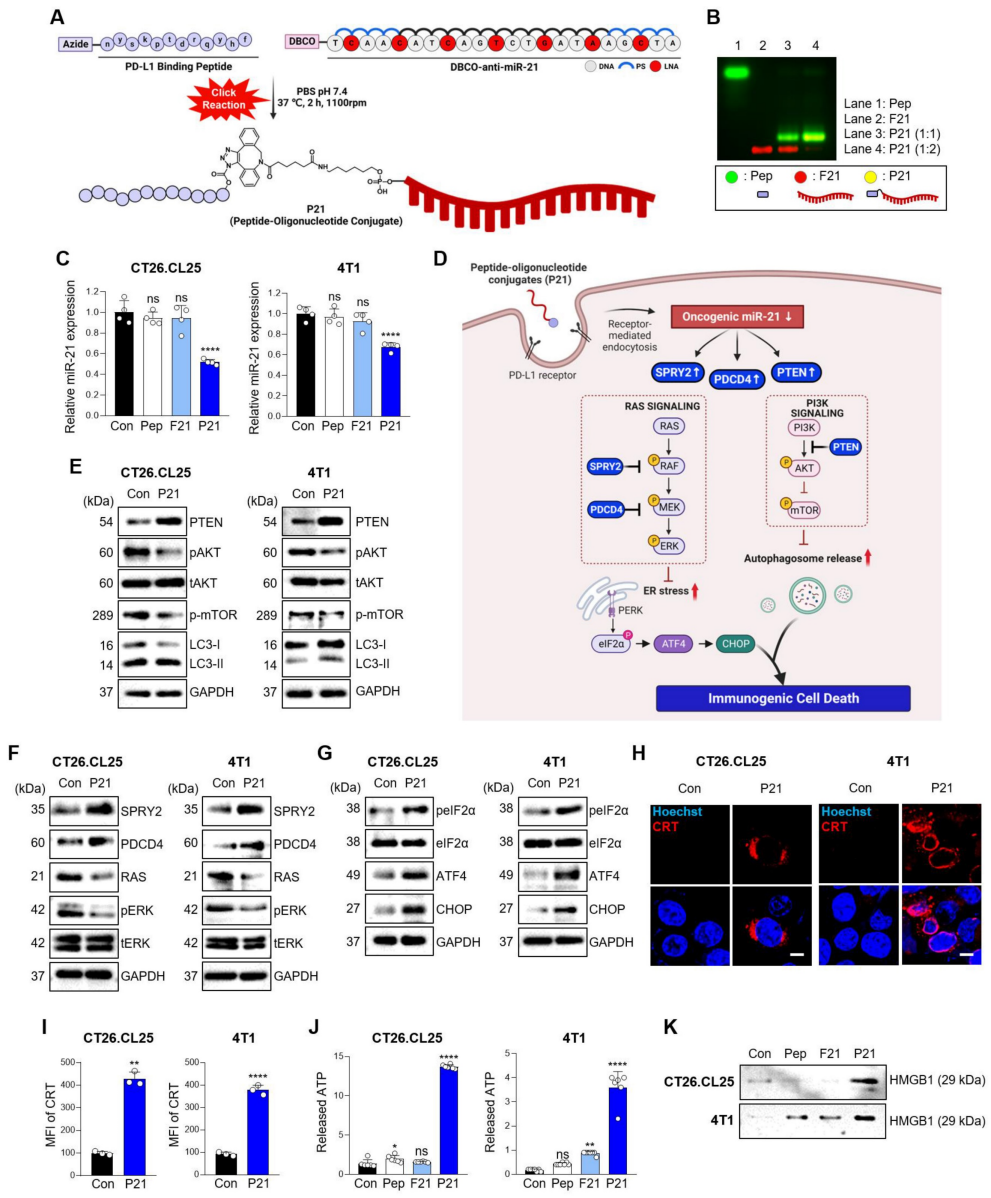
** Inhibition of miR-21 triggers ICD via activation of tumor suppressor genes in PD-L1^high^ cancer cells. (A)** Schematic diagram of the overall synthesis strategy for P21. **(B)** Representative agarose gel images showing the characterization of synthesized P21. **(C)** Relative miR-21 expression levels measured by RT-qPCR in CT26.CL25 and 4T1 cells after treatment with Pep (150 nM), F21 (150 nM), or P21 (150 nM) for 18 h. All samples were normalized to U6 expression (*n* = 4/group). **(D)** Schematic of P21 antitumor properties in cancer cells. **(E)** Western blot analysis of PTEN, p-AKT, p-mTOR, and LC3-II abundance in CT26.CL25 (left) and 4T1 (right) cells after treatment with P21 (150 nM) for 24 h. **(F)** Western blot analysis of SPRY2, PDCD4, and RAS/ERK abundance in CT26.CL25 (left) and 4T1 (right) cells after treatment with P21 (150 nM) for 24 h. **(G)** Western blot analysis of p-elF2a, t-elF2a, ATF4, and CHOP abundance in CT26.CL25 (left) and 4T1 (right) cells after treatment with P21 (150 nM) for 4 h. **(H)** Representative confocal images of CRT (red) expression on the surface of CT26.CL25 (left) and 4T1 (right) cells following treatment with P21 (150 nM) for 20 h (scale bar = 50 μm; *n* = 3/group). The nuclei were stained with Hoechst 33342 (blue). **(I)** The expression levels of CRT were measured by flow cytometry. Data are presented as the relative mean fluorescence intensity against the control (*n* = 3/group). **(J)** Relative expression of released ATP from CT26.CL25 and 4T1 cell lines after treatment with Pep (150 nM), F21 (150 nM), or P21 (150 nM) for 24 h (*n* = 7/group). **(K)** Western blot analysis of extracellular HMGB1 abundance in CT26.CL25 and 4T1 cell lysates after treatment with Pep (150 nM), F21 (150 nM), or P21 (150 nM) for 24 h (*n* = 3/group). Data are presented as the mean ± SD (*p < 0.05, **p < 0.01, ****p < 0.0001). Statistical significance was calculated by **(C, J)** one-way ANOVA followed by Tukey's multiple comparisons test and **(I)** two-tailed unpaired Student's t-test.

We previously synthesized and prepared P21 using a copper-free click reaction with a 2:1 ratio of azidoacetylated-modified PD-L1-binding peptide to Diarylcyclooctyne (DBCO)-functionalized anti-21 (F21), demonstrating an optimal conjugation efficiency of over 90% **(Figure 1A and 1B)** 22. To assess the impact of P21 on the cellular response, we selected CT26.CL25 colon cancer cells, and 4T1 breast cancer cells due to their notable overexpression of miR-21 and the PD-L1 receptor on the cell membranes compared to normal colonic epithelium cells NCM460 **(Figure S1A and S1B)**. P21 exhibited superior cellular uptake and significantly reduced oncogenic miR-21 levels in both cell types compared to the other treatment groups **(Figure 1C, Figure S1D, and S1E)**.

However, the reduction in oncogenic miR-21 via cellular P21 uptake showed no lethal toxicity in either cell type **(Figure S1C)**. Although miR-21 is a well-known oncogene crucial in promoting cancer cell survival and resistance to programmed cell death, elucidating the mechanisms underlying the anticancer effects of miR-21 inhibition remains a considerable challenge 23-25. Therefore, we initially investigated whether the P21-mediated downregulation of oncogenic miR-21 leads to tumor suppressor gene activation, specifically *PTEN* and *PDCD4*, and their subsequent downstream signals. We hypothesized that inhibiting miR-21 could activate PTEN-mediated autophagy and PDCD4-related ER stress, collectively enhancing the release of damage-associated molecular patterns (DAMPs) and triggering ICD induction **(Figure 1D)** 26. As anticipated, P21 treatment significantly increased the abundance of PTEN protein in both cancer cell lines and effectively suppressed phosphorylated-AKT (p-AKT) and phosphorylated-mTOR (p-mTOR) levels, which are downstream signals of PTEN **(Figure 1E)**. These findings strongly suggest that P21 directly regulates the PTEN/AKT/mTOR signaling pathway involved in autophagy. Additionally, we observed enhanced expression of autophagy markers, LC3-II. Together, these results suggest that P21 can drive autophagy via the PTEN/AKT/mTOR signaling pathway.

Furthermore, miR-21, a crucial oncogene in cancer treatment, inhibits the negative regulators of RAS/MEK/ERK signaling by suppressing the expression of PDCD4 and SPRY2 27, 28. As shown in **Figure 1F**, P21 upregulates SPRY2 and PDCD4 protein abundance, leading to a marked downregulation of phosphorylated-ERK1/2 (p-ERK1/2), which is recognized as a downstream signal of RAS. Importantly, the inhibition of RAS signaling can elicit ER stress, exhibiting calreticulin (CRT) exposure on the plasma membrane during cancer immunotherapy, resulting in ICD 29, 30. To further explore this aspect, we evaluated whether P21 could enhance ER stress signaling via activation of the PERK/eIF2α/ATF4/CHOP axis in both cell lines. As expected, the expression levels of phosphorylated-elf2-α (p-elf2-α), ATF4, and CHOP increased, supporting the notion that P21 activates ER stress responses by regulating RAS activation **(Figure 1G)**.

Triggering excessive ER stress induces autophagy in tumor cells, which ultimately elicits cell death 31. To confirm this, we investigated whether P21 could provoke ICD by promoting the release of DAMPs from both cancer cell lines. This involved measuring the surface-exposed CRT and the release of high-mobility group box 1 (HMGB1) and adenosine triphosphate (ATP). Our observations showed that P21 promoted the translocation of CRT and enhanced its expression on the cell surface **(Figure 1H)**. P21-triggered CRT exposure on the surface of cancer cells was also confirmed using flow cytometry **(Figure 1I and S1F)**. Notably, in both CT26.CL25 and 4T1 cells, P21 treatment markedly increased the secretion of ATP and HMGB1 into the supernatants compared to the PBS-treated control group (Con) **(Figure 1J and 1K)**. These results suggest that P21 acts as an ICD inducer by promoting CRT translocation and enhancing the production of extracellular DAMPs, such as HMGB1 and ATP. Importantly, ICD-derived DAMPs can stimulate antigen-presenting cells (APCs), such as macrophages and DCs, and influence CD8^+^ T cell function.

### P21-induced DAMP release enhances DC phagocytic activity and maturation

To explore the interaction between P21 and DCs, we isolated BMDCs from BALB/c mice, induced their differentiation into immature DCs, and verified their differentiation status using flow cytometry with an anti-CD11c antibody **(Figure S1G)**. We observed increased expression of miR-21 and PD-L1 in DCs, confirming that they were target subsets of P21 **(Figure S1H and S1I)**. As expected, P21 was successfully internalized by DCs and significantly suppressed oncogenic miR-21 levels compared to the other treatment groups without inducing cellular toxicity **(Figure S1J-L)**.

**Figure 2:**
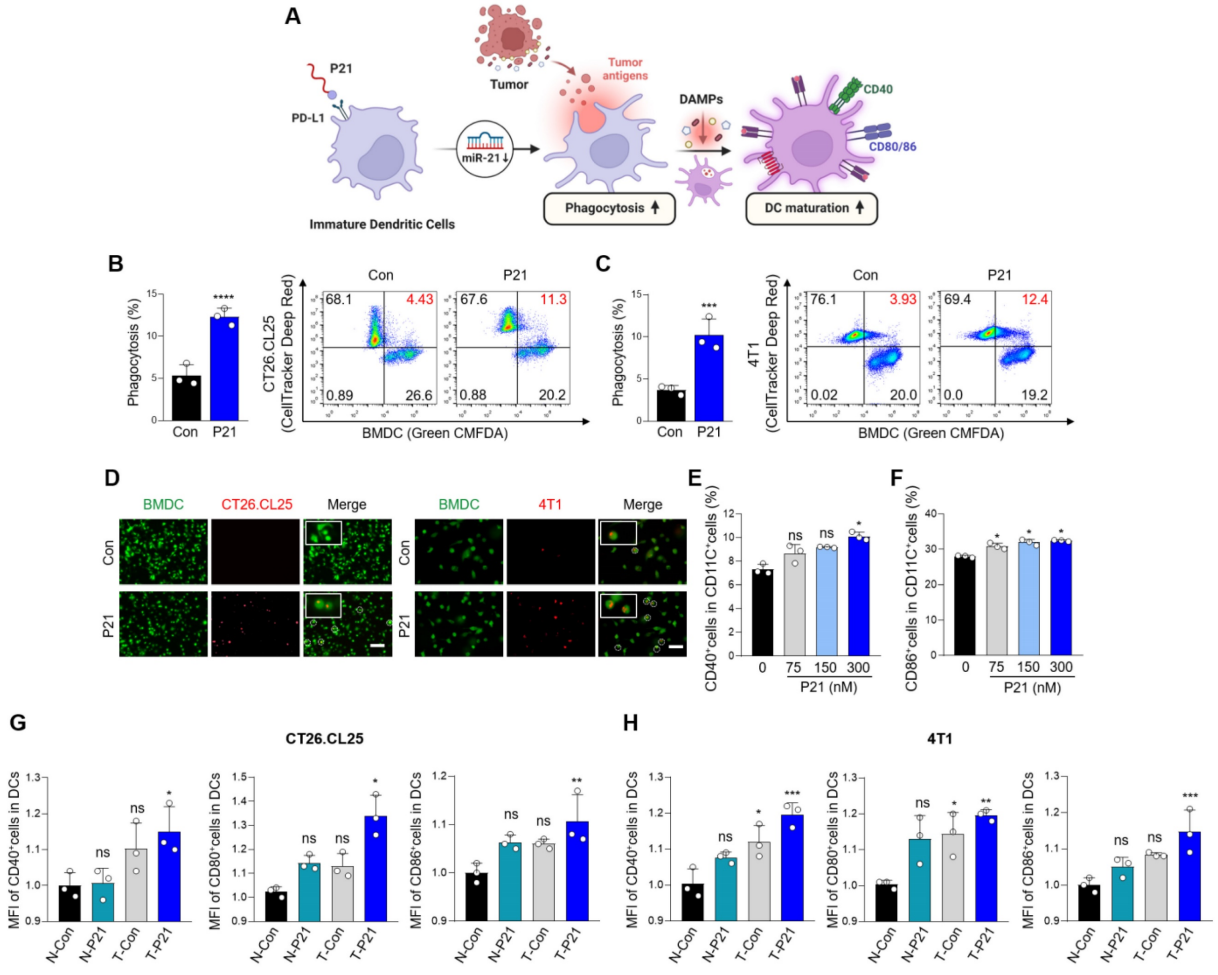
** P21-induced DAMP exposure enhances the phagocytic activity and maturation of DCs. (A)** Schematic of P21 antitumor properties in DCs. **(B and C)** Representative flow cytometry analysis of DC phagocytic activity. BMDCs and BMDCs after P21 (150 nM) treatment or no treatment for 24 h were co-cultured with untreated or P21-treated CT26.CL25 and 4T1 cells for an additional 24 h (*n* = 3/group). Phagocytosis (%) was calculated based on the total number of BMDCs. **(D)** Representative microscopic images of phagocytosis assays were performed using pHrodo-SE-labeled CT26.CL25 and 4T1 cells (red) against green CMFDA-labeled BMDCs (green) (scale bar = 100 μm; *n* = 3/group). **(E and F)** Expression of DC maturation markers (CD11C^+^CD40^+^ or CD86^+^) measured by flow cytometry. Data are presented as the relative MFI values against the control (*n* = 3/group). **(G and H)** Expression of DC maturation markers (CD11C^+^CD40^+^, CD80^+^, or CD86^+^) measured by flow cytometry. Data are presented as the relative mean fluorescence intensity against the control (*n* = 3/group). BMDCs after P21 (300 nM) treatment or no treatment for 24 h co-cultured with untreated or P21-treated CT26.CL25 and 4T1 cells for an additional 24 h (N: non-treated cancer cells, T: P21-treated cancer cells) (*n* = 3/group). Data are presented as the mean ± SD (*p < 0.05, **p < 0.01, ***p < 0.001, ****p < 0.0001). Statistical significance was calculated by **(B, C)** two-tailed unpaired Student's t-test and (E-H) one-way ANOVA followed by Tukey's multiple comparisons test.

DCs are highly efficient APCs that activate antitumor CD8^+^ T cells by phagocytosing dying tumor cells and presenting co-stimulatory molecules, such as CD80 and CD86 **(Figure 2A)**. In particular, DAMPs induced by P21 and released from dying tumors trigger signals that promote the recognition of cancer cells by DCs. In this regard, P21 treatment can significantly boost the phagocytic capacity of DCs, enabling more efficient capture of tumor antigens. To validate this, CT26.CL25 and 4T1 cells were treated with P21 and subsequently co-cultured with BMDCs for 2 h. The proportion of phagocytic cells substantially increased in the P21-treated group **(Figure 2B and 2C)**. Similar observations were observed microscopically for both cell lines **(Figure 2D)**. We also assessed the augmentation of macrophage phagocytosis. As anticipated, P21 treatment increased the phagocytic capacity of bone marrow-derived macrophages (BMDMs) against CT26.CL25 and 4T1 cells **(Figure S2A-D)**. These results are consistent with our previous study, demonstrating P21's ability to repolarize M2-like tumor-associated macrophages (TAMs) into M1-like TAMs through miR-21 inhibition in BMDMs 22. Collectively, our findings indicate that P21 can enhance the phagocytic activity of DCs and macrophages by promoting ICD in tumor cells.

Overexpression of miR-21 is associated with the immature state of BMDCs, whereas its depletion fosters DC maturation, resulting in an increased production of pro-inflammatory cytokines 14. The presence of mature DC markers (CD11C^+^CD40^+^ or CD86^+^) increased in a concentration-dependent manner upon P21 treatment **(Figure 2E, 2F, S2E and S2F)**. Beyond P21's direct influence on miR-21 inhibition and DC maturation, the augmented phagocytic capacity of DCs toward cancer cells can also contribute to DC maturation. Therefore, we investigated whether P21 efficiently stimulates DC maturation through ICD induction. The proportion of mature DCs was evaluated by co-culturing supernatants from non-treated or P21-treated CT26.CL25 and 4T1 cells with BMDCs. As predicted, the administration of P21 to only DCs or cancer cells slightly enhanced DC maturation (CD11C^+^CD40^+^, CD80^+^, or CD86^+^) compared with the untreated group **(Figure 2G and 2H)**. However, we found that P21-treated DCs co-incubated with P21-treated cancer cells exhibited a synergistic effect on the proportion of mature DCs compared with the individual treatment groups. Taken together, our findings underscore the role of P21-induced DAMP signaling in augmenting the phagocytic activity of APCs. Furthermore, P21-mediated mature DCs exhibit heightened expression of co-stimulatory molecules, enhancing antigen presentation to promote T cell activation.

### PD-L1-dependent tumor accumulation of P21 induces an antitumor immune response in CT26.CL25 tumor-bearing mice

**Figure 3:**
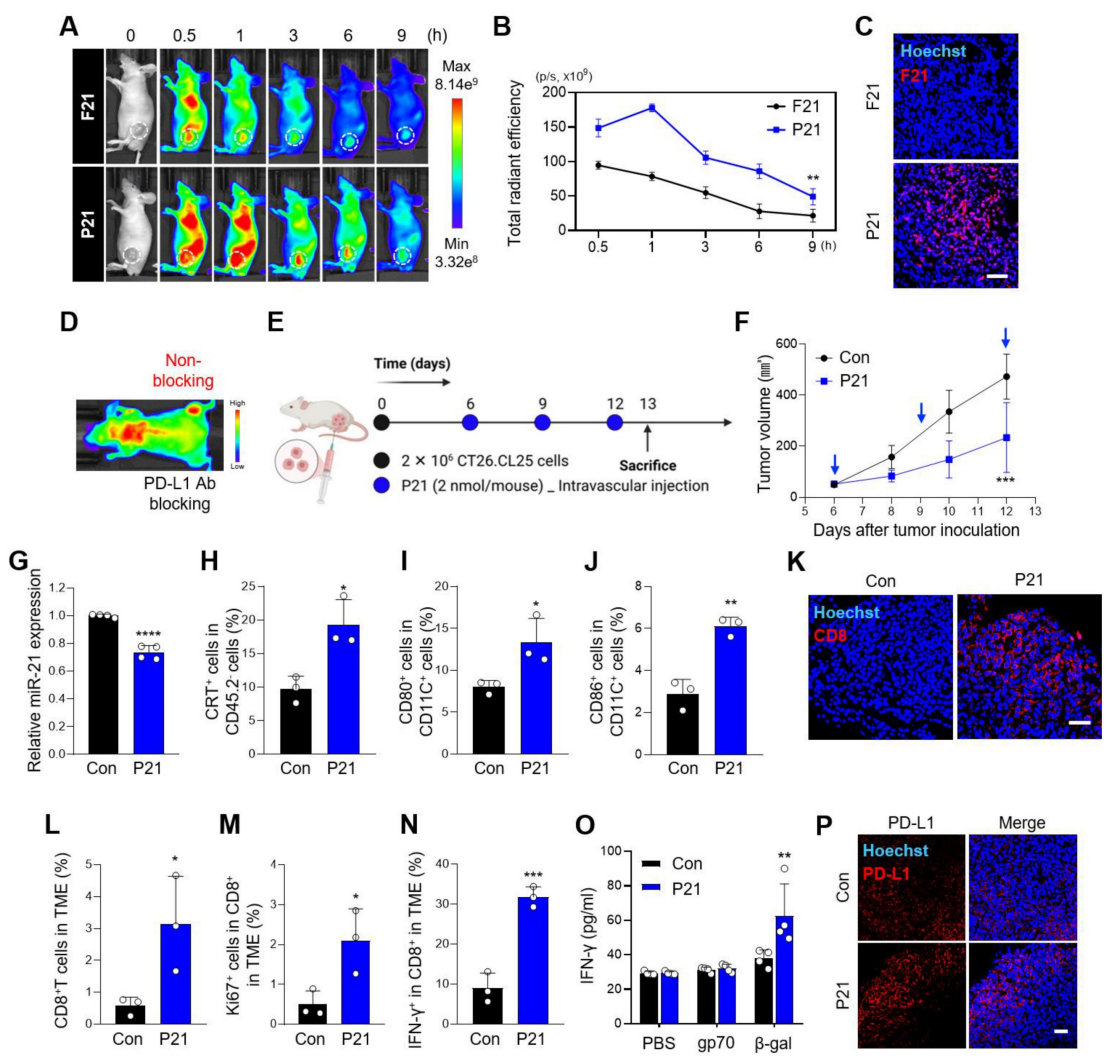
** P21 utilization promotes tumor-targeting and therapeutic efficacy in a CT26.CL25 tumor-bearing mice model. (A)** Whole-body fluorescence images in CT26.CL25 tumor-bearing nude mice at 0.5, 1, 3, 6, and 9 h after tail vein injection of Cy5-labeled P21 or F21 (4 nmol/mouse) and **(B)** quantification of fluorescence intensities (*n* = 3/group). **(C)** Representative fluorescence images of tumor tissues injected with Cy5-labeled P21 or F21 (scale bar = 50 μm; *n* = 3/group). The nuclei were stained with Hoechst 33342 (blue). **(D)** IVIS fluorescence imaging of mice bearing bilateral CT26.CL25 tumors. Tumors in the left flank were pre-blocked with anti-PD-L1 antibody; Cy5-labeled P21 (4 nmol/mouse) was then injected into the tail vein. **(E)** Schematic of P21 administration to CT26.CL25 tumor-bearing mice. **(F)** Tumor growth curves of CT26.CL25-bearing mice were monitored for 13 days after tumor inoculation (*n* = 9/group; P21: 2 nmol/mouse). **(G)** Relative miR-21 expression measured by RT-qPCR in tumor tissues. All samples were normalized to U6 expression (*n* = 4/group). **(H)** Expression of CRT-positive cancer cells (CD45.2^‑^CRT^+^) measured by flow cytometry (*n* = 3/group). **(I and J)** Expression of tumor-infiltrating mature DCs (CD11c^+^CD80^+^ or CD86^+^) measured by flow cytometry (*n* = 3/group). **(K)** Representative immunofluorescence images of tumor-infiltrating CD8^+^ T cells after 13 days of tumor inoculation (scale bar = 50 μm; *n* = 4-5/group). The nuclei were stained with Hoechst 33342 (blue). **(L)** Proportion of total T cells (CD45.2^+^CD3^+^CD8^+^) and **(M and N)** activated CD8^+^ T cells (CD45.2^+^CD3^+^CD8^+^Ki67^+^ or IFN-γ^+^) (*n* = 3/group). **(O)** Splenocytes were stimulated with the β-gal peptide, or gp70 peptide for 48 h, and the amount of IFN-γ was evaluated by ELISA (*n* = 4/group). **(P)** Representative immunofluorescence images of PD-L1 expression (red) in tumor tissues (scale bar = 50 μm; *n* = 3/group). The nuclei were stained with Hoechst 33342 (blue). Data are presented as the mean ± SD (*p < 0.05, **p < 0.01, ***p < 0.001, ****p < 0.0001). Statistical significance was calculated by **(B, F, O)** two-way ANOVA with Sidak's multiple comparison test and (G, H, I, J, L, M, N) two-tailed unpaired Student's t-test.

Prior to evaluating the antitumor effects of P21 *in vivo*, we investigated its tumor-targeting ability in a CT26.CL25 tumor-bearing mouse model. Once, the average tumor volume reached approximately 150-200 mm^3^, F21 or P21 was administered (4 nmol/mouse) via tail vein injection. F21 decreased rapidly within 1 h and exhibited only weak fluorescence at each time point compared with the P21 group. In contrast, P21 sustainably retained fluorescence for 9 h and exhibited enhanced uptake by tumor tissue within 30 min through PD-L1 receptor-mediated endocytosis. This suggests that P21 was tumor-specifically delivered in the CT26.CL25-tumor bearing mouse model **(Figure 3A and 3B)**. As shown in **Figure. 3C**, the relative fluorescence level of F21 labeled with Cy5 (red) in the tumor tissues was higher in the P21-treated group than in the F21-treated group.

Next, we analyzed the delivery basics of P21 in a bilateral murine tumor model. After localized administration of anti-PD-L1 to tumors in the left flank of mice, we measured the fluorescence intensity of Cy5-labeled P21 injected through the tail vein. To prevent anti-PD-L1 from spreading systemically and affecting the contralateral tumors, P21 was injected within a 1 h window. Interestingly, tumors that were blocked with anti-PD-L1 showed low fluorescence intensity, whereas those in the unblocked right flank exhibited a higher accumulation of P21 fluorescence intensity **(Figure 3D)**. Taken together, these observations provide evidence that P21 can successfully target tumor regions in a PD-L1 receptor-dependent manner.

We then verified the therapeutic efficacy of P21 in a CT26.CL25 tumor-bearing mouse model. When the average tumor volume reached 40-50 mm^3^, P21 was administered via the tail vein every 3 days for a total of three injections at a dose of 0.78 mg/kg (2 nmol/mouse; **Figure 3E**). P21 strongly suppressed tumor growth compared to that in the PBS-treated control group **(Figure 3F)**. We also observed that P21 considerably inhibited miR-21 expression compared to the control group **(Figure 3G)**. Tumor tissues collected on day 13 showed elevated translocation of CRT to the cell surface in the P21 treatment group, suggesting that P21 plays a key role as an ICD inducer **(Figure 3H and S3A)**.

DC maturation is promoted by P21, however, is synergistically enhanced by DAMP signals released from P21-treated tumors. Consistent with the *in vitro* results, the proportion of mature DCs (CD11C^+^CD40^+^, CD80^+^, or CD86^+^) in tumor tissues and tumor-draining lymph nodes (TDLNs) was highly upregulated compared to that in the control group **(Figure 3I, 3J, and Figure S3B-E)**. In addition, P21 elicited higher levels of total immune cell populations (CD45.2^+^) than in the control group, which correlated with an increase in total and activated CD8^+^ T cells **(Figure S3F)**. Moreover, confocal and flow cytometry analyses revealed that the P21 treatment group showed markedly amplified tumor-infiltrating CD8^+^ T cells in the TME **(Figure 3K)** and increased proportions of CD8^+^ T cells (CD45.2^+^CD3^+^CD8^+^; **Figure 3L and S3G**). The frequency of the proliferation marker Ki67^+^ was also significantly increased in CD8^+^ T cells of the P21 group compared to the control group **(Figure 3M)**. Collectively, we confirmed not only the superior therapeutic efficacy of P21 but also its ability to effectively heighten antitumor immunity through DC maturation and CD8^+^ T cell activation.

### Combined P21 and anti-PD-L1 therapy effectively amplifies antitumor immunity in the TME

Next, we analyzed the antigen-specific IFN-γ secretion in splenocytes from CT26.CL25 tumor-bearing mice. Compared to the control treatment group, P21 markedly increased IFN-γ secretion, a positive indicator of a high response rate to PD-1/PD-L1 blockades **(Figure 3N, 3O, and S3H)**. Consistent with our expectations, IFN-γ-mediated PD-L1 expression in the TME was significantly upregulated in the P21 group **(Figure 3P and S3I)**.

**Figure 4:**
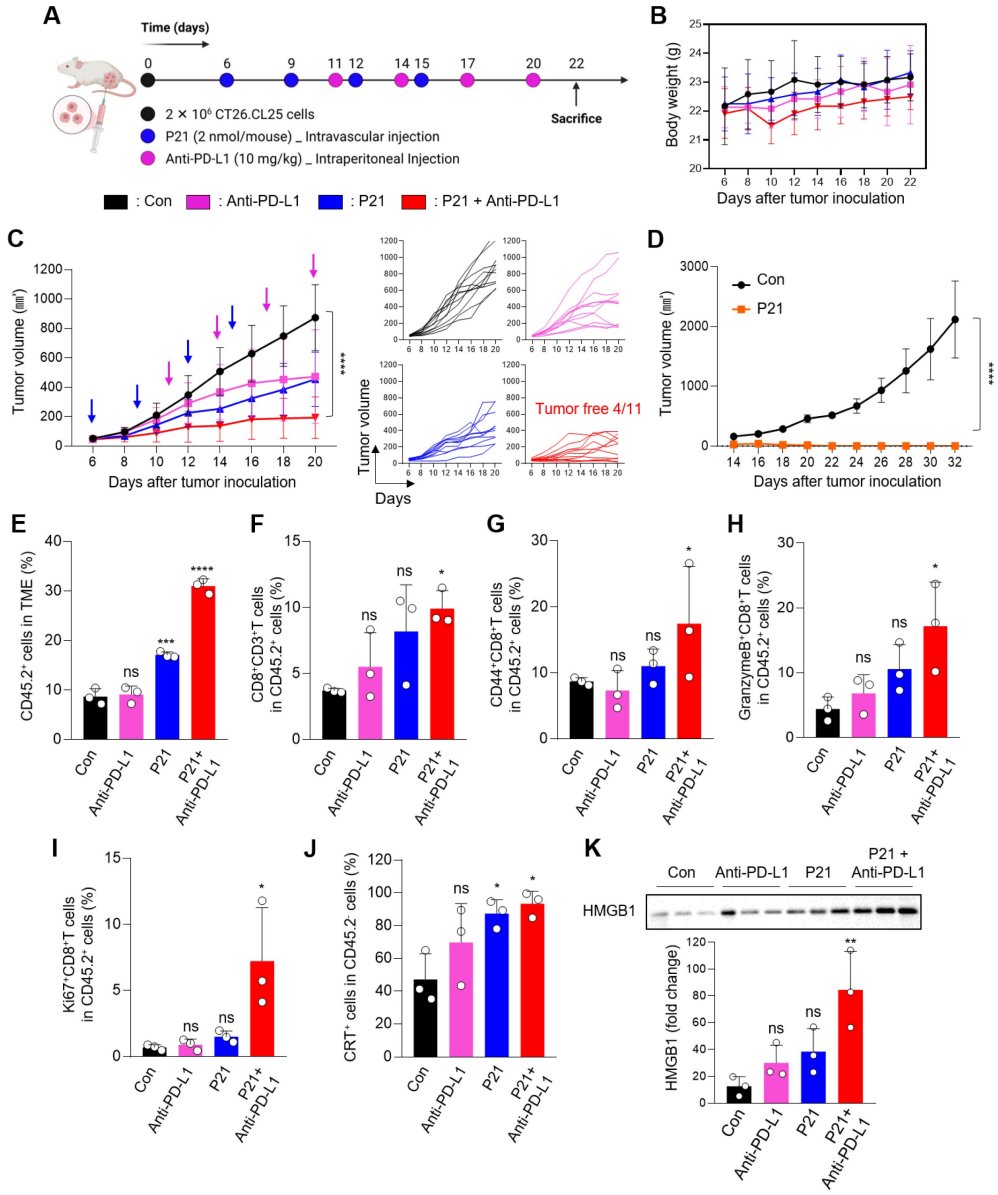
** Combination therapy with P21 and anti-PD-L1 effectively amplifies antitumor immunity in the TME. (A)** Schematic of the combination of P21 and anti-PD-L1 therapy in the CT26.CL25 tumor-bearing mice model. **(B)** Body weight changes in mice for each group. **(C)** Tumor growth curves for CT26.CL25-bearing mice over 22 days after tumor inoculation (anti-PD-L1:10 mg/kg, P21:2 nmol/mouse) (*n* = 11/group). **(D)** Tumor re-challenge of the tumor-free mice from the combination group with control sex- and age-matched mice (*n* = 4/group). Tumor-free mice were re-challenged in the contralateral flank with 2 × 10^6^ CT26.CL25 cells one month after complete regression of the primary tumor. **(E)** Representative flow cytometry analysis of the total immune cells (CD45.2^+^) in the TME (*n* = 3/group). **(F)** Representative proportions of the total T cell (CD45.2^+^CD3^+^CD8^+^) and (G-I) CD8^+^ T cell activation markers (CD45.2^+^CD3^+^CD8^+^CD44^+^, Granzyme B^+^, or Ki67^+^) (*n* = 3/group). **(J)** Expression of CRT-positive cancer cells (CD45.2^‑^CRT^+^) measured by flow cytometry (*n* = 3/group). **(K)** Western blot analysis of HMGB1 abundance in tumor tissues (*n* = 3/group). Data are presented as the mean ± SD (*p < 0.05, **p < 0.01, ***p < 0.001, ****p < 0.0001). Statistical significance was calculated by **(B, C, D)** two-way ANOVA with Sidak's multiple comparison test and **(E-K)** one-way ANOVA followed by Tukey's multiple comparisons test.

Therefore, to evaluate the synergistic therapeutic effects of combined P21 and anti-PD-L1 therapy, each drug was injected into the CT26.CL25 tumor-bearing mouse model according to the schedule presented in **Figure 4A** (0.78 mg/kg P21 and 10 mg/kg anti-PD-L1). Compared to all monotherapy groups, combined P21 and anti-PD-L1 exerted greater tumor growth inhibition without significant changes in body weight **(Figure 4B, 4C, and S4A)**. The combination treatment group also exhibited complete tumor suppression in 36.4% (4/11) of the mice, highlighting the synergistic antitumor effects in the TME. After one month of rest, tumor-free mice were re-injected with the same CT26.CL25 tumor cells in the opposite flank, and tumor growth was monitored for 32 days. These mice exhibited resistance to the re-challenged CT26.CL25 tumor cells, in contrast, age-matched control mice were sensitive to CT26.CL25 cells **(Figure 4D)**. These results indicate that P21 induced a robust and long-lasting immune response that effectively prevented tumor recurrence.

Subsequently, to evaluate the immunomodulatory effect of P21, we analyzed the immune cell infiltrates of the TME. As expected, an increased population of overall immune cells (CD45.2^+^) on live cells was observed in the P21 and combination treatment groups **(Figure 4E and S4B)**. In particular, the proportion of tumor-infiltrating CD8^+^ T cells (CD45.2^+^CD3^+^CD8^+^) was markedly increased in the TME of the combination treatment group **(Figure 4F)**. Furthermore, CD44, a CD8^+^ T cell activation marker, was more abundant in the combination treatment group (CD45.2^+^CD3^+^CD8^+^CD44^+^; **Figure 4G**). Consistent with this result, the combination treatment group exhibited a marked increase in Granzyme B and Ki67 expression in the TME (CD45.2^+^CD3^+^CD8^+^Granzyme B^+^ or Ki67^+^), which serve as markers for the effector functions and proliferative activity of activated T cells, respectively **(Figure 4H and 4I)**.

We also observed elevated levels of the P21-induced ICD markers CRT and HMGB1 in the P21 monotherapy and combination therapy groups **(Figure 4J, 4K, and S4C)**. Notably, the P21 monotherapy group exhibited high expression of ICD markers, even though the analysis was conducted 7 days after the final P21 treatment. Moreover, the increased number of LC3-II-positive cells in the tumor tissue suggested that P21-induced reactivation of PTEN led to autophagy, ultimately activating ICD **(Figure S4D)**.

### Combined P21 and anti-PD-L1 therapy exhibits a synergistic antimetastatic effect

**Figure 5:**
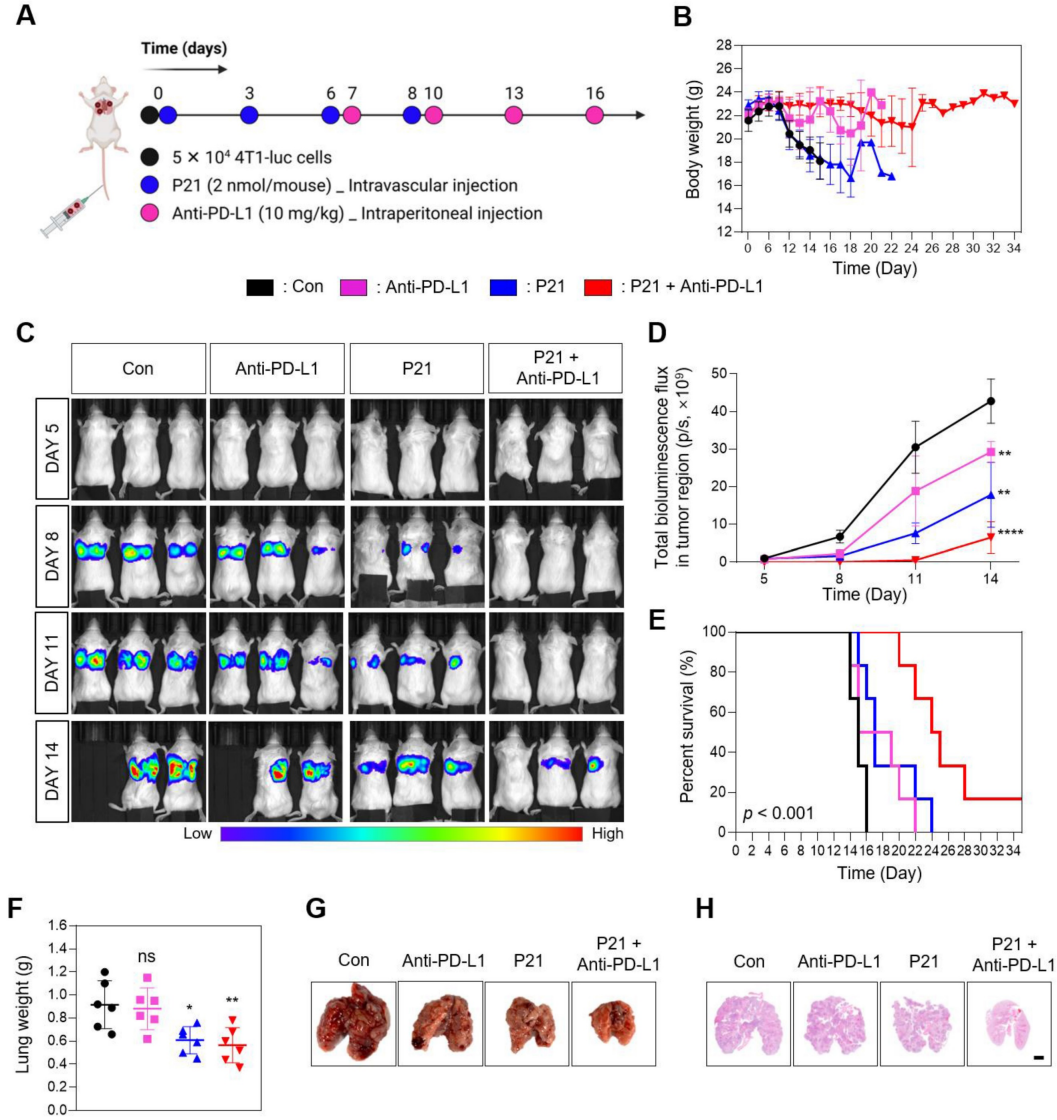
** Combined P21 and anti-PD-L1 therapy achieves a synergistic antimetastatic effect. (A)** Schematic of the experimental design for the P21 and anti-PD-L1 combined therapy in a 4T1 lung metastatic model. **(B)** Body weight changes of mice for each group (*n* = 6/group). **(C)** Bioluminescence images for metastatic lung cancer growth and **(D)** total bioluminescence flux measured via IVIS Lumina on days 5, 8, 11, and 14 (*n* = 3/group). **(E)** Survival curves for metastatic lung cancer mice during treatment (*n* = 6/group). **(F)** Weights of lungs excised from the 4T1 lung metastatic model at day 15 after the indicated treatments. **(G)** Representative lung tissue images and **(H)** sections of whole lungs extracted from mouse tumor metastasis models 15 days after the indicated treatments (scale bar = 2000 μm; n = 3/group). Data are presented as the mean ± SD (*p < 0.05, **p < 0.01, ***p < 0.001, ****p < 0.0001). Statistical significance was calculated by **(D, F)** one-way ANOVA followed by Tukey's multiple comparisons test and **(E)** long-rank Mantel-Cox text.

Considering the significant role of miR-21 in tumor metastasis, we assessed the therapeutic efficacy of a combination approach (P21 plus anti-PD-L1) in a lung metastasis model via intravenous injection of 4T1-luc cells. Subsequently, the mice were treated with anti-PD-L1, P21, or anti-PD-L1 + P21 (0.78 mg/kg F21 and 10 mg/kg anti-PD-L1) according to the treatment schedule presented in **Figure 5A**. We co-administered P21 and tumor cells on day 0 to delay cancer progression compared to the other groups. The anti-PD-L1 monotherapy exhibited no significant effects on lung tumor growth. Moreover, in the PBS-treated control and anti-PD-L1 monotherapy groups, bright bioluminescent signals were detected from day 8 onwards and gradually increased over time, indicating a progressive pattern of metastatic growth **(Figure 5B and 5D)**. This resulted in overall physical deterioration, weight loss, and premature death **(Figure 5C)**. In contrast, P21 monotherapy and combination treatment with anti-PD-L1 elicited low bioluminescent signals 14 days after tumor inoculation. In particular, the combination treatment group exhibited no bioluminescence signal until day 11, and only a weak signal by day 14. Thus, combination treatment prolonged the survival rate of mice **(Figure 5E)**, highlighting the superior therapeutic efficacy of combining P21 and anti-PD-L1 in metastatic lung cancer models.

The results for the excised lungs confirmed that the lungs in the P21 and combination treatment groups had lower weights than those in the other groups, remaining close to their original lung size **(Figure 5F and 5G)**. Histological examination of lung tissues using hematoxylin and eosin (H&E) staining further demonstrated that the combination group had relatively healthy and normal lung structures with few pulmonary metastatic nodules. Meanwhile, the control and anti-PD-L1 monotherapy groups exhibited markedly decreased lung quality with numerous pulmonary metastatic nodules **(Figure 5H)**. Taken together, these results demonstrate that combination therapy efficiently inhibits metastatic tumor progression and provides a novel anti-metastatic strategy.

## Discussion

More than 60% of the human protein-coding genes are predicted to be selectively regulated by miRNAs.^32^ In particular, the robust regulation of miRNAs in the transcriptome enables basic and translational studies of miRNAs for the clinical management of cancer.^33^ However, since miRNAs play a crucial role in regulating the immune response in the TME through interactions between several immune and cancer cells, the potential of miRNA therapeutics in the TME should be carefully evaluated to develop effective and safe miRNA-based anti-cancer treatment strategies 34, 35.

In this study, we elucidated the intricate relationship between miR-21 and the mechanism for anticancer immunity mechanism associated with inhibiting miR-21. For the first time, we showed that diminishing miR-21 levels by targeting PD-L1^high^ tumors triggers tumor ICD. This process is activated by PTEN-related autophagy and PDCD4-associated ER stress. While previous studies have primarily focused on validating the depletion of miR-21 and the restoration of tumor suppressor genes, our findings emphasize that reactivating these suppressor genes leads to the activation of explicit mechanisms involving autophagy and ER stress. These mechanisms contribute to ICD by promoting DC maturation and inducing potent CD8^+^ T cell responses, leading to strong anticancer effects. These results indicate why miR-21 inhibition exhibits a milder inhibitory effect on cancer cell proliferation *in vitro* than on tumor growth* in vivo*. Furthermore, immune activation within the TME, resulting from reduced miR-21 expression, enhances sensitivity to immune checkpoint blockade therapy.

Despite the success of miRNA therapeutics in preclinical studies, no miRNA-based therapy has been approved for cancer treatment. To date, only two miRNA-based drugs (an LNA-based oligonucleotide inhibitor of miR-155 for hematopoietic malignancies, NCT02580552; miR-16 targomiR for the treatment of malignant pleural mesothelioma, NCT02369198) have entered the clinical phase for cancer patients resistant to traditional treatments or those with untreatable tumors 36-38. Hence, for miRNA therapeutics to become available for cancer treatment, targeted delivery must be achieved to minimize off-target effects 39.

A crucial aspect of targeting the TME *in vivo* involves covalently conjugating specific ligands—PD-L1 binding peptides—to anti-miR21 inhibitors. This strategy enhances the uptake of oligonucleotide drugs at specific tumor sites. The interaction between these peptides and PD-L1 receptors leads to the endocytosis of peptide-conjugated anti-miR21 drugs. While the PD-L1 receptor is typically absent in normal tissues, IFN-γ can induce PD-L1 expression in nearly any nucleated cell 40. However, miR-21 expression is carefully regulated in normal cells to remain below a specific threshold 4, 41, preventing the indiscriminate modulation of its target genes. Therefore, the peptide-conjugated anti-miR21 drug selectively targets cells within the TME, including cancer cells, DCs, and macrophages that exhibit elevated levels of both PD-L1 and miR-21 expression, thus, eliciting remarkable antitumor effects.

To pave the way for the utilization of miRNAs in primary cancer therapeutics in the near future, it is imperative to comprehensively explore their mechanisms of action, including their ability to elicit antitumor immune responses and therapeutic effects, which must be comprehensively characterized, while also considering the potential unpredictable immunogenicity associated with miRNA therapeutics. Therefore, we suggest that the employment of targeted delivery of anti-miR-21 against PDL1^high^ TME might be a promising synergistic approach for tumor cancer immunotherapy.

## Materials and Methods

### Materials

The N-terminal azidoacetylated-modified PD-L1-binding peptide, *Asn-Tyr-Ser-Lys-Pro-Thr-Asp-Arg-Gln-Tyr-His-Phe* (N_3_-nyskptdrqyhf, d-form), was synthesized by Peptron (Republic of Korea). 5′-DBCO-functionalized anti-miR-21-5p comprising DNA (DBCO-5′-TCAACATCAGTCTGATAAGCTA-3′), Cy5-labeled anti-miR-21, DBCO-anti-miR-21 with backbone modifications (DBCO-5′-*T*C*AA*C*A*TCAGTCTGATA*AG*C*TA*-3′, underlined identify locked nucleic acids (LNAs) and italics represent phosphorothioate (PS)), and Cy5-labeled anti-miR-21 with backbone modifications, were purchased from Bioneer (Republic of Korea).

### Cell lines

CT26.CL25 (mouse colorectal carcinoma), 4T1 (mouse breast cancer), and 4T1-luc (mouse breast cancer) cell lines were obtained from the American Type Culture Collection (ATCC, USA) and cultured and maintained in Roswell Park Memorial Institute (RPMI)-1640 (Welgene, Republic of Korea) supplemented with 10% fetal bovine serum (FBS; Gibco, USA) and 1% antibiotic-antimycotic (Gibco, USA). The NCM460 (normal human colonic epithelial; INCELL, USA) cell line was cultured and maintained in Dulbecco's modified eagle medium (DMEM)-high glucose (Hyclone, USA) supplemented with 10% FBS and 1% antibiotic-antimycotic solution.

Bone marrow (BM) cells were generated and maintained according to previously reported methods 42. Briefly, BM cells were isolated from the hind legs of 7-week-old male BALB/c mice and seeded in 100-mm culture dishes to remove unwanted cells. To generate BMDMs, the cells were collected and re-plated in 100-mm petri dishes at a density of 3.5 × 10^6^ cells with 20 ng/mL murine macrophage colony-stimulating factor (M-CSF; Peprotech, USA) on day 1. The cells were treated with 20 ng/mL M-CSF on days 2 and 3, and the cell medium was replaced with a growth medium containing 20 ng/mL M-CSF on days 4 and 6. To stimulate M2 polarization, cells were treated with interleukin-4 (IL-4; Peprotech, USA) for 24 h. For differentiation to BMDCs, cell media were replaced with growth media containing 20 ng/mL granulocyte-macrophage colony-stimulating factor (GM-CSF), 20 ng/mL IL-4, and 0.1% β-mercaptoethanol on days 3 and 5.

### Flow cytometry

For flow cytometry analysis, CT26.CL25, 4T1, BMDMs, and BMDCs were seeded in 35-mm glass-bottom dishes at a density of 3 × 10^5^ cells/well and incubated overnight at 37 °C. The following day, single-cell suspensions were harvested and pre-blocked with Fc blocker (BD Pharmingen, USA, clone 2.4G2, #553142) for 15 min at 4 °C. To observe the cell surface expression level, single cells were labeled by fluorescence-conjugated antibodies for 30 min at 4 °C. The collected cells were washed three times with Dulbecco's phosphate-buffered saline (DPBS; Welgene, Republic of Korea) to suspend the pellets as single cells. All samples were analyzed using a CytoFLEX flow cytometer (Beckman Coulter, USA) and the FlowJo (v10) software. The following antibodies used in this study were obtained from BioLegend (USA): APC-anti-F4/80 (clone BM8, #123116), FITC-anti-CD11b (clone M1/70, #101205), APC-anti-CD11c (clone N418, #117309), APC-anti-PD-L1 (clone 10F.9G2, Cat #: 124311), and APC-anti-PD-L1 (clone 29E.2A3, #329707).

### *In vitro* cellular uptake

CT26.CL25 and 4T1 cells were seeded in 35-mm glass-bottom confocal dishes at a density of 2 × 10^5^ cells/well, while BMDCs were seeded at a density of 1 × 10^6^ cells/well, and incubated overnight at 37 °C. The following day, the cells were treated with saline, Cy5-labeled DBCO-functionalized F21, or Cy5-labeled P21 at an equivalent concentration of 150 nM in a serum-free medium for 6 h. After incubation, the cells were washed in DPBS three times to remove the nonspecific binding and fixed with 4% formaldehyde for 10 min. The nuclei were stained with Hoechst 33342 (Invitrogen, USA) for 10 min at room temperature. Fluorescence imaging was performed using the CS SP8 confocal laser microscope (Leica TCS SP5; Leica, Germany).

### In vitro cytotoxicity assay

CT26.CL25, 4T1 cells, and BMDCs were seeded in 96-well plates at a density of 5 × 10^3^ cells/well and incubated for 24 h at 37 °C. After stabilization, the cells were treated with saline, peptide (Pep), F21, or P21 at a final concentration of 150 nM. After 24 h, 10 μL of Cell Counting Kit-8 (CCK-8; Dojindo Laboratory, Japan) solution was added to each well, and the absorbance was measured using a microplate reader (SpectraMax 34, Molecular Devices, USA) at 450 nm.

### Quantitative reverse transcription-polymerase chain reaction (qRT-PCR)

To analyze miR-21 expression, CT26.CL25, 4T1 cells, and BMDCs were seeded in 35-mm culture dishes at a density of 2 × 10^5^ cells/well and incubated overnight at 37 °C. After stabilization, cells were treated with saline, Pep, F21, or P21 at a final concentration of 150 nM F21 in a serum-free medium for 18 h. Total RNA was isolated using a miRNeasy mini kit (Qiagen, Germany) and reverse-transcribed to cDNA using Mir-X miRNA First Strand Synthesis (Takara Bio, Japan). Finally, qPCR was performed with the StepOnePlus Real-Time PCR System (Applied Biosystems, USA) using TOPreal™ SYBR Green qPCR Kits (Enzynomics, Republic of Korea). The relative expression of miR-21 was normalized to U6 RNA and assessed using the 2^-ΔΔCt^ method. All qRT-PCR procedures for miRNAs were performed according to the manufacturer's instructions. The following primers were used in this study 43: U6 forward, 5′-CTCGCTTCGGCAGCACA-3′; miR-21 forward, 5′-AGACTAGCTTATCAGACTGA-3′; and universal reverse, 5′-GTGCAGGGTCCGAGGT-3′. All primers were supplied by Cosmo Genetech (Republic of Korea).

### Western blot analysis

CT26.CL25 and 4T1 cells were seeded in 6-well plates at a density of 1 × 10^5^ cells/well and incubated overnight at 37 °C. After stabilization, the cells were treated with saline or P21 (150 nM) in a serum-free medium for the indicated times. Next, the cells were lysed in RIPA buffer (Thermo Fisher Scientific, USA) supplemented with 1% protease/phosphatase inhibitor cocktail for 20 min on ice, and the cell supernatants were collected by centrifugation at 12,000 × g for 20 min. The protein concentration was calculated using the BCA Protein Assay Kit (Bio-Rad, USA). Equal amounts of protein were electrophoresed on a 12% sodium dodecyl sulfate-polyacrylamide gel electrophoresis (SDS-PAGE) gel for 90 min at 100 V and transferred onto nitrocellulose membranes. The membrane blots were then blocked with 5% skim milk for 1 h and incubated with the primary antibodies overnight at 4 °C. The next day, the membranes were washed three times with TBS-T (pH 7.4, 20 mM Tris, 150 mM NaCl, and 0.1% Tween 20) for 10 min and incubated with appropriate secondary antibodies at room temperature for 1 h. After washing with TBS-T, protein signals were visualized using ECL substrate (Bio-Rad, USA) and detected with chemiluminescence (iBright; Invitrogen, USA). The antibodies used in this study were as follows: PTEN (CST, USA, #9959; dilution 1:1000), PDCD4 (Abcam, UK, ab51495; 1:1000), phospho-Akt (CST, USA, #9271S; 1:500), total-Akt (CST, USA, #9272; 1:1000), phospho-mTOR (CST, USA, #2971; 1:1000), LC3B (CST, USA, #2775; 1:1000), Spry2 (CST, USA, #14954; 1:1000), RAS (CST, USA, #33197; 1:1000), phospho-p44/42 MAPK (Erk1/2) (CST, USA, #9101; 1:1000), p44/42 MAPK (Erk1/2) (CST, USA, #9102; 1:1000), phopho-elF2α (CST, USA, #9721; 1:1000), elF2α (CST, USA, #9722; 1:1000), ATF4 (Santa Cruz Biotechnology, USA, sc-390063; 1:500), CHOP (Santa Cruz Biotechnology, USA, sc-7351; 1:500), GAPDH (GeneTex, USA, GTX100118; 1:1000), anti-Rabbit IgG-HRP antibody (GeneTex, USA, GTX213110-01; 1:2000), and anti-mouse IgG-HRP antibody (GeneTex, USA, GTX213111-01; 1:2000).

### Detection of ICD-induced markers (DAMPs)

To identify ICD-induced DAMPs, CT26.CL25 and 4T1 cells were seeded in 6-well plates at a density of 2 × 10^5^ cells/well. After stabilization, both cell lines were treated with saline, Pep, F21, or P21 at a final concentration of 150 nM F21 in a serum-free medium for 24 h. The surface exposure level of CRT was detected by staining single cells with an Alexa 647-conjugated CRT antibody (Abcam, UK, ab196159; 1:100), and mean fluorescent intensity (MFI) was measured using a CytoFLEX flow cytometer. Subsequently, for fluorescence analysis of CRT exposure, single cells were fixed with 4% formaldehyde for 10 min and stained with Alexa 647-conjugated CRT antibody overnight at 4 °C. The nuclei were stained with Hoechst 33342 (Invitrogen, USA) for 10 min at room temperature. The CRT surface expression levels were observed using a CS SP8 confocal laser microscope. To detect extracellular HMGB1 in the cell supernatants, the collected culture supernatants were quantitatively analyzed by western blotting. To quantify the release level of ATP in the cell supernatants, the collected culture supernatant was processed using a commercial ENLITEN ATP Assay System Bioluminescence Detection Kit (Promega, USA) according to the manufacturer's instructions under the same experimental conditions.

### Phagocytosis assay

For the *in vitro* phagocytosis assay, 2 × 10^5^ BMDMs or 1.2 × 10^6^ BMDCs were stained with CellTracker Green CMFDA (1 μM; Thermo Fisher Scientific, USA) for 30 min at room temperature and incubated with or without P21 (150 nM) for 24 h. Subsequently, 1 × 10^5^ CT26.CL25 or 4T1 cells were stained with CellTracker Deep Red (1 μM; Thermo Fisher Scientific, USA) for 30 min at room temperature and incubated with or without P21 (150 nM) for 24 h. Cancer cells, either untreated or treated with P21, were co-cultured with BMDMs or BMDCs at a ratio ranging from 1:2 to 1:4 at 37 °C for 2 h. The cells were then washed with DPBS three times to suspend the pellets as single cells. All samples were analyzed using a CytoFLEX flow cytometer.

For microscopic evaluation of phagocytosis, 2 × 10^5^ BMDMs or 1.2 × 10^6^ BMDCs labeled with CMFDA were seeded in confocal dishes and treated with or without P21 (150 nM) for 24 h. Subsequently, 1 × 10^5^ CT26.CL25 or 4T1 cells were stained with pHrodo-SE (Thermo Fisher Scientific, USA) for 30 min at room temperature and treated with or without P21 (150 nM) for 24 h. Next, untreated or P21-treated cancer cells were co-cultured with BMDMs or BMDCs for 2 h at 37 °C at a ratio of 1:2 to 1:4. The cells were then harvested and washed with pH 10 PBS to remove cells that had not been engulfed, and fluorescence images were captured by fluorescence microscopy (Evos M5000; Thermo Fisher Scientific, USA).

### *In vitro* DC maturation

For the DC maturation assay, BMDCs were treated with saline or P21 at a final concentration of 300 nM F21 in a serum-free medium for 24 h. Immature BMDCs were then co-cultured with cell supernatants containing cellular DAMPs from CT26.CL25 or 4T1 cells pretreated with P21 (150 nM) for 24 h. The cells were then harvested, stained with fluorescence-conjugated antibodies for 30 min at 4 °C, collected, and washed three times with DPBS to suspend the pellets as single cells. All samples were analyzed using a CytoFLEX flow cytometer. The following antibodies used in this study were purchased from BioLegend (USA): APC-anti-CD11c (clone N418, #117309), PE-anti-CD11c (clone N418, #117307), APC-anti-CD40 (clone3/23, #124611), FITC-anti-CD86 (clone GL-1, #105005), and APC anti-CD80 (clone 16-10A1, #104713).

### *In vivo* biodistribution study

*In vivo* biodistribution imaging was performed following subcutaneous injection of 6-week male BALB/c mice with 2 × 10^6^ CT26.CL25 cells in the left flank. When the average tumor volume reached 150-200 mm^3^, the mice were intravenously injected with F21 or P21 (4 nmol/mouse) labeled with Cy5 via the tail vein. Fluorescent signals were monitored for 9 h and captured using an IVIS II Lumina device (Lumina Series III; PerkinElmer, USA). For *ex vivo* fluorescence imaging, mice were sacrificed 9 h after the last injection, and tumor tissues and major organs were collected for visualization.

### *In vivo* tumor models

Mice were purchased from Orient Bio (Republic of Korea). All the animal studies were performed in a specific pathogen-free animal facility at the Korea Institute of Science and Technology (KIST) according to the Institutional Animal Care and Use Committee (IACUC) guidelines of KIST (approval number: KIST-2022-04-072). Six-week-old male BALB/c mice were subcutaneously injected with 2 × 10^6^ CT26.CL25 cells in the left flank. When the average tumor volume reached approximately 40-60 mm^3^, all mice were randomly divided into two treatment groups (Con, P21). 0.78 mg/kg P21 was intravenously injected three times on days 6, 9, and 12. The injection interval was determined by the serum stability of P21, in which the stability of chemical backbone-modified P21 was maintained for 72 h in mouse serum 22.

The antitumor efficacy was evaluated by sacrificing mice 13 days after the last P21 injection. Then, tumor tissues and TDLNs were harvested and processed into single cells using a MACS Tumor Dissociation Kit (Miltenyi Biotec, USA) according to the manufacturer's instructions. The single cells were counted and pre-blocked with Fc blocker (BD Pharmingen, USA, clone 2.4G2, #553142) for 15 min at 4 °C to avoid nonspecific antibody binding. Next, the single cells were labeled by fluorescence-conjugated antibodies for 30 min at 4 °C. The collected cells were washed three times with DPBS to resuspend the pellets as single cells. All samples were analyzed using a CytoFLEX flow cytometer with the FlowJo (v10) software. The following antibodies used in this study were obtained from BioLegend (USA): PE-anti-CD45.2 (clone 104, #109807), FITC-anti-CD3 (clone 17A2, #100203), APC-anti-CD8 (clone 53-6.7, #100711), PE-anti-Ki67 (clone 11F6, #151209), FITC-anti-IFN-γ (clone XMG1.2, #505805), FITC-anti-CD44 (clone IM7, #103005), and FITC-anti-Granzyme B (clone GB11, #515403).

We analyzed the following populations in tumor tissues: (i) CRT-positive cancer cells (CD45.2^‑^CRT^+^), (ii) maturated DCs (CD45.2^+^CD11c^+^CD80^+^, CD86^+^, or CD40^+^), (iii) total CD8-positive T cells (CD45.2^+^CD3^+^CD8^+^), (iv) proliferative T cells (CD45.2^+^CD3^+^CD8^+^Ki67^+^), and (v) activated T cells (CD45.2^+^CD3^+^CD8^+^IFNγ^+^, CD44^+^, or Granzyme B^+^). To enhance the reliability of the results, animals were randomly selected into groups for flow cytometry and histological analyses.

For combination therapy with an anti-PD-L1 antibody (anti-PD-L1), 6-week-old male BALB/c mice were subcutaneously injected with 2 × 10^6^ CT26.CL25 cells into the left flank. After the tumors had grown for six days to reach an approximate volume of 40-60 mm^3^, all mice were randomly divided into four treatment groups: saline, anti-PD-L1, P21, and P21 + anti-PD-L1 combination. 0.78 mg/kg P21 was intravenously injected a total of four times on days 6, 9, 12, and 15. In some experiments, mice were intraperitoneally injected with 10 mg/kg anti-PD-L1 monoclonal antibody (BioXCell, USA, #BE0101) for four times on days 11, 14, 17, and 20. Moreover, a tumor re-challenge experiment was conducted in mice that had eliminated the primary tumor after combination treatment. That is, mice were re-injected with 2 × 10^6^ CT26.CL25 cells in the opposite flank 4 weeks after complete remission. The control mice were age and sex-matched. Tumor volumes and body weights were monitored every other day, calculated with a caliper, and calculated using the following formula: tumor volume = (width^2^ × length)/2.

To analyze the metastatic model, BALB/c mice were intravenously injected with 5 × 10^4^ 4T1-luc cells. All mice were then randomly divided into four treatment groups: saline, anti-PD-L1, P21, and P21 + anti-PD-L1 combination. P21 was intravenously injected a total of four times on days 0, 3, 6, and 9. The anti-PD-L1 (10 mg/kg) was intraperitoneally injected four times every 3 days on days 7, 10, 13, and 16. Lung tissues were extracted on day 17 after tumor inoculation, and the bioluminescence intensity of the tissues was analyzed using an IVIS II Lumina device.

### Histological analysis

For immunofluorescence staining of CD8, PD-L1, and LC3B, tumor tissues were obtained from the CT26.CL25 model on the day after the final injection and fixed in a 4% paraformaldehyde solution. Tumor tissues were embedded in paraffin and cut into 8-μm thick sections using a rotary microtome (Accu-cut SRM200 Rotary Microtome, Japan). The sections were then pre-blocked with a blocking solution (1% BSA and 0.5% Triton X-100 in PBS) for 15 min at room temperature to prevent nonspecific binding. After washing with TBS-T buffer for three times, the sections were stained with APC fluorescent-conjugated anti-CD8, APC fluorescent-conjugated anti-PD-L1, or Alexa 647 anti-LC3B (Abcam, UK, ab22583) at 4 °C overnight and nuclei were stained with Hoechst 33342 for 10 min. All images were analyzed using a TCS SP8 confocal laser microscope.

For H&E staining, the metastatic lung tissue sections were stained with an H&E staining kit (Abcam, UK, ab245880) according to the manufacturer's protocol and observed by A BX51 Optical microscope (Olympus, USA).

### Statistical analysis

All statistical analyses were performed using Prism 8 (GraphPad) and presented as the mean ± standard deviation (SD). Statistical significance between groups was calculated using student's *t*-tests or one-way (or two-way) ANOVA followed by Tukey's multiple comparisons test.

## Supplementary Material

Supplementary figures.

## Abbreviations

miR-21: miRNA-21
TME: Tumor microenvironment
P21: Anti-miR-21 via a peptide conjugate
PTEN: Phosphatase and tensin homolog
PDCD4: Programmed cell death protein 4
DCs: Dendritic cells
BMDCs: Bone marrow-derived DCs
anti-miR-21: Inhibitor of oncogenic miR-21
ICD: Immunogenic cell death
ER: Endoplasmic reticulum
DBCO: Diarylcyclooctyne
LNAs: Locked nucleic acids
PS: Phosphorothioate
ATCC: American type culture collection
RPMI: Roswell park memorial institute
FBS: Fetal bovine serum
DMEM: Dulbecco's modified eagle medium
BM: Bone marrow
M-CSF: Murine macrophage colony-stimulating factor
IL-4: Interleukin-4
GM-CSF: Granulocyte-macrophage colony-stimulating factor
F21: DBCO-functionalized anti-21
Pep: Peptide
SDS-PAGE: Sodium dodecyl sulfate-polyacrylamide gel electrophoresis
DAMP: Damage-associated molecular patterns
CRT: Calreticulin
HMGB1: High-mobility group box 1
ATP: Adenosine triphosphate
TDLNs: Tumor-draining lymph nodes
H&E: Hematoxylin and eosin
p-AKT: Phosphorylated AKT
p-mTOR: Phosphorylated mTOR
p-ERK1/2: Phosphorylated-ERK1/2
APCs: Antigen-presenting cells
TAMs: Tumor-associated macrophages
