# Supplementary material for: Targeted delivery of anti-miRNA21 sensitizes PD-L1high tumor to immunotherapy by promoting immunogenic cell death
Source: Theranostics. 2024 Jun 17;14(10):3777–92. doi: 10.7150/thno.97755 (PMC11234275; doi:10.7150/thno.97755)
Supplement: Supplementary file 1 — Supplementary figures. [file thnov14p3777s1.pdf]

Supplementary Information

Targeted delivery of anti-miRNA21 sensitizes PD-L1^{high} tumor to immunotherapy by promoting immunogenic cell death

Eun Hye Kim[1,2], Jiwoong Choi[1], Hochung Jang[1,3], Yelee Kim[1,2], Jong Won Lee[1,4], Youngri Ryu[1,2], Jiwon Choi[1,5], Yeonho Choi[5], Sung-Gil Chi[2], Ick Chan Kwon[1,4], Yoosoo Yang[1,3] and Sun Hwa Kim[1,4]**

[1]Medicinal Materials Research Center, Biomedical Research Division, Korea Institute of Science and Technology (KIST), Seoul 02792, Republic of Korea

[2]Department of Life Sciences, Korea University, Seoul 02841, Republic of Korea

[3]Division of Bio-Medical Science and Technology, KIST School, University of Science and Technology, Seoul 02792, Republic of Korea

[4]KU-KIST Graduate School of Converging Science and Technology, Korea University, Seoul 02841, Republic of Korea

[5]Department of Bioengineering, Korea University, Seoul 02841, Republic of Korea

*Corresponding author:

Yoosoo Yang, Ph.D. (Y. Yang)

Tel: +82-2-958-6655; fax: +82-2-958-5909; e-mail: ysyang@kist.re.kr

Sun Hwa Kim, Ph.D. (S.H. Kim)

Tel: +82-2-958-6639; fax: +82-2-958-5909; e-mail: sunkim@kist.re.kr

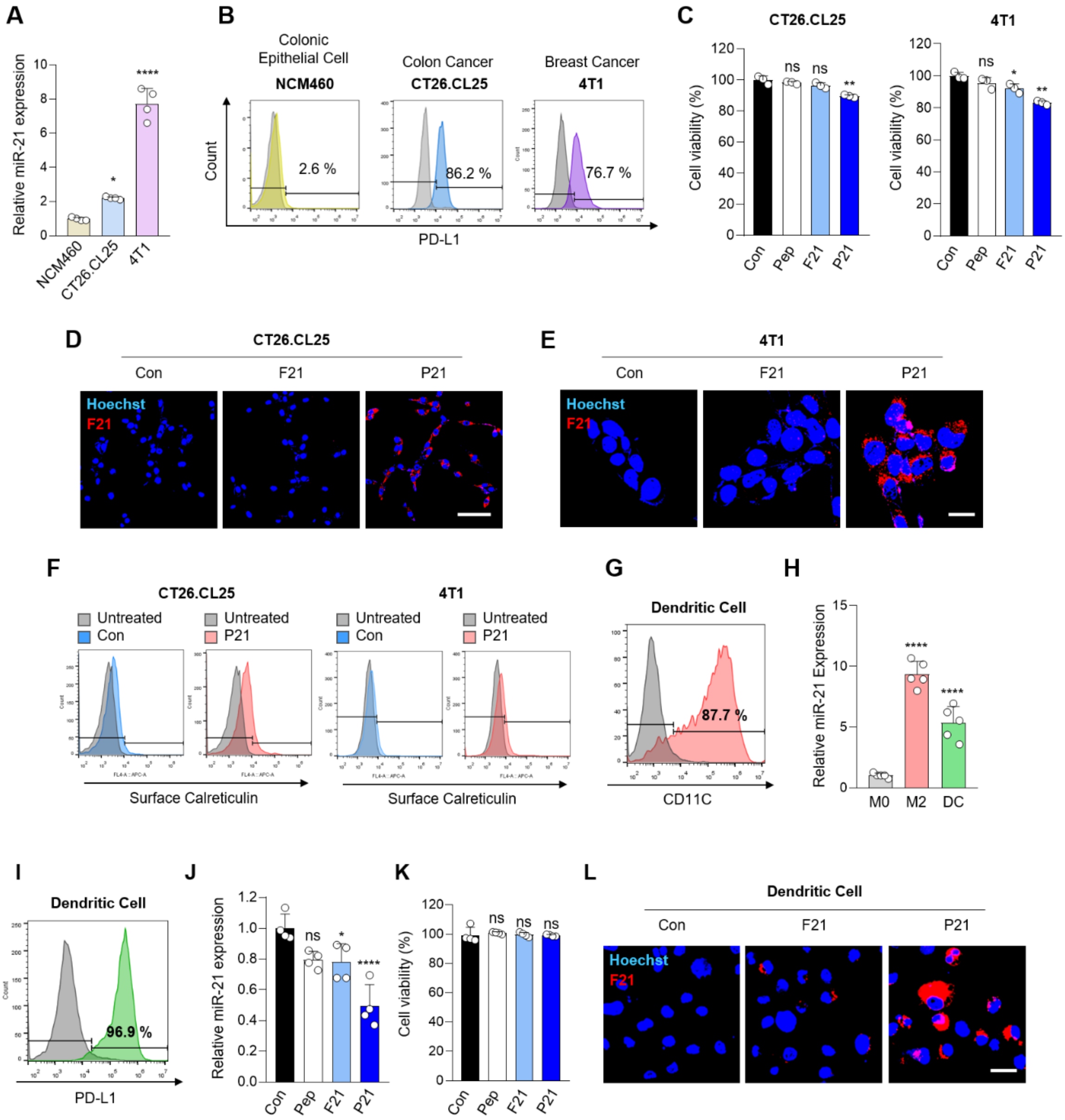

Figure S1. (A) Relative miR-21 expression levels measured by RT-qPCR in NCM460, CT26.CL25 and 4T1 cell lines. All samples were normalized to U6 expression (n = 4/group). (B) Expression of PD-L1 in indicated cancer cell lines measured by flow cytometry (grey: isotype control). (C) Viability of CT26.CL25 (left) and 4T1 (right) cell lines following treatment with Pep (300 nM), F21 (300 nM), or P21 (300 nM) for 24 h (n = 3/group). (D) Representative confocal images of uptake by CT26.CL25 cell lines after treatment with F21 (300 nM) or P21 (300 nM) for 6 h. The nuclei were stained with Hoechst 33342 (blue) (scale bar = 200 μm; n = 3/group). (E) Representative confocal images of uptake by 4T1 cell lines after treatment with F21 (300 nM) or P21 (300 nM) for 6 h. The nuclei were stained with Hoechst 33342 (blue) (scale bar = 50 μm; n = 3/group). (F) Expression of CRT measured by flow cytometry (grey: isotype control). (G) Representative histograms of BMDCs differentiation with anti-CD11c antibody. (H) Relative miR-21 expression was measured by RT-qPCR in M0, M2, and DCs. All samples were normalized to U6 expression (n = 4/group). (I) Expression of PD-L1 in BMDCs measured by flow cytometry. (J) Relative miR-21 expression was measured by RT-qPCR in BMDCs after treatment with Pep (150 nM), F21

(150 nM), or P21 (150 nM) for 18 h. All samples were normalized to U6 expression (*n* = 4/group). (K) Viability of BMDCs after treatment with Pep (300 nM), F21 (300 nM), or P21 (300 nM) for 24 h (*n* = 4/group). (L) Representative confocal images of uptake by BMDCs after treatment with F21 (300 nM) or P21 (300 nM) for 6 h. The nuclei were stained with Hoechst 33342 (blue) (scale bar = 50 μm; *n* = 3/group). Data are presented as the mean ± SD (*$p < 0.05$, **$p < 0.01$, ****$p < 0.0001$). Statistical significance was calculated by (A, C, H, J, K) one-way ANOVA followed by Tukey's multiple comparisons test.

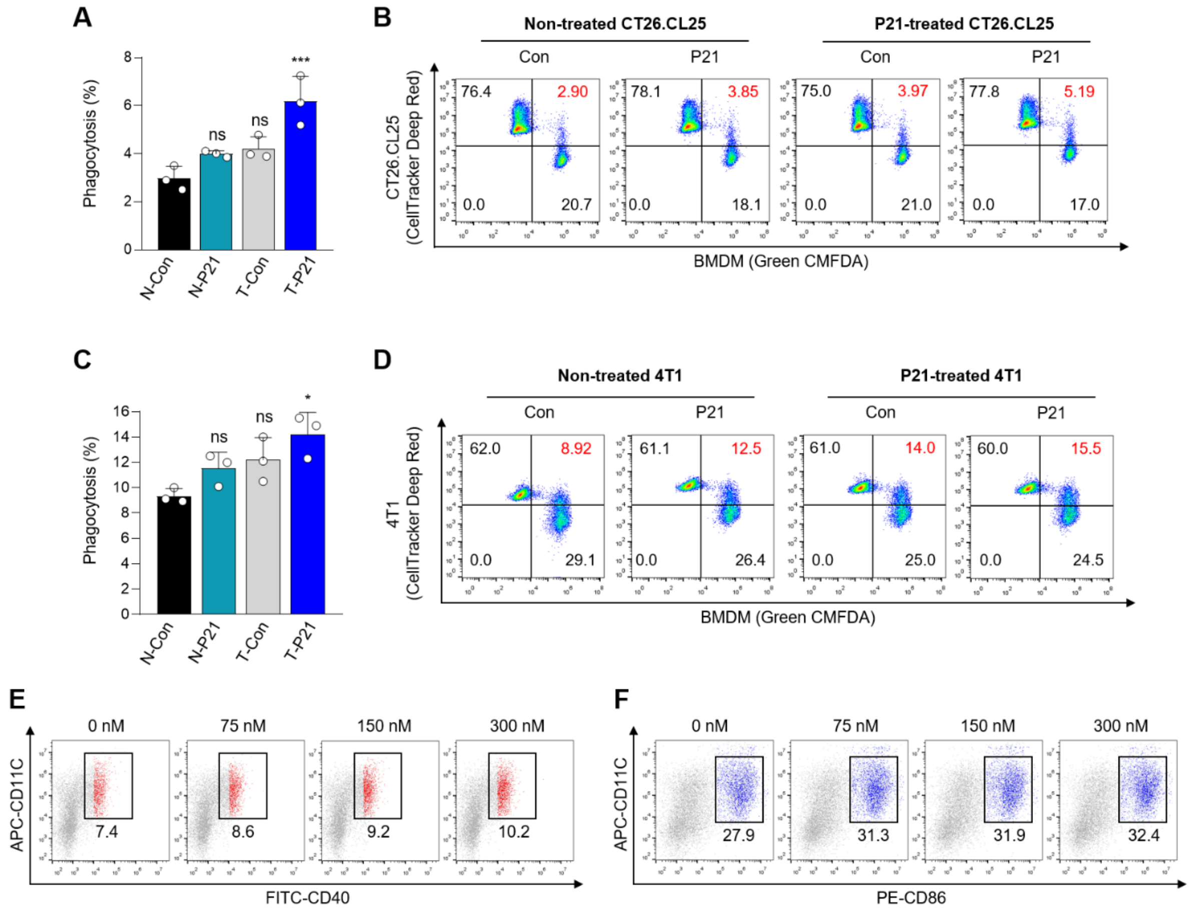

Figure S2. (A–D) Representative flow cytometry analysis of macrophage phagocytic activity. BMDMs and BMDMs after P21 (150 nM) treatment or no treatment for 24 h were co-cultured with untreated or P21-treated CT26.CL25 and 4T1 cells for an additional 24 h (N: non-treated cancer cells, T: P21-treated cancer cells) (n = 3/group). Phagocytosis (%) was calculated based on the total number of BMDMs. (E and F) Expression of DC maturation markers ($CD11C^{+}CD40^{+}$ or $CD86^{+}$) measured by flow cytometry. Data are presented as the relative mean fluorescence intensity against the control (n = 4/group). Data are presented as the mean ± SD (*p < 0.05, ***p < 0.001). Statistical significance was calculated by (A, C) one-way ANOVA followed by Tukey’s multiple comparisons test.

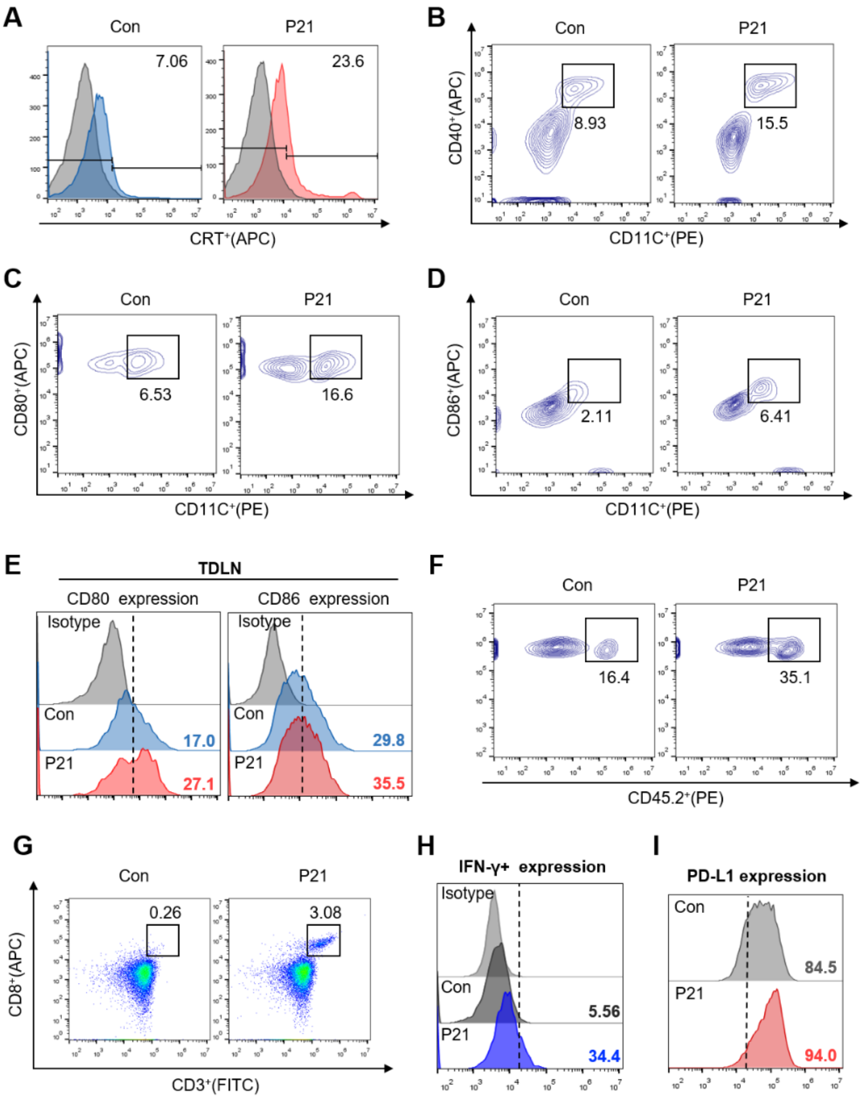

Figure S3. (A) Expression of CRT on tumor tissues measured by flow cytometry (CD45.2$^-$CRT$^+$) (grey: isotype control). (B–D) Representative flow cytometry data showing tumor-infiltrating mature DCs (CD11c$^+$CD40$^+$ or CD80$^+$ or CD86$^+$). (E) Representative flow cytometry data showing mature DCs (CD11c$^+$CD80$^+$ or CD86$^+$) in TDLN. (F) Representative flow cytometry analysis of the total immune cell (CD45.2$^+$) proportion in the TME. (G) Representative flow cytometry data of tumor-infiltrating CD8$^+$ T cells (CD45.2$^+$CD3$^+$CD8$^+$). (H) Representative flow cytometry analysis of IFN-γ$^+$-expressing and (I) PD-L1-expressing cells in the TME.

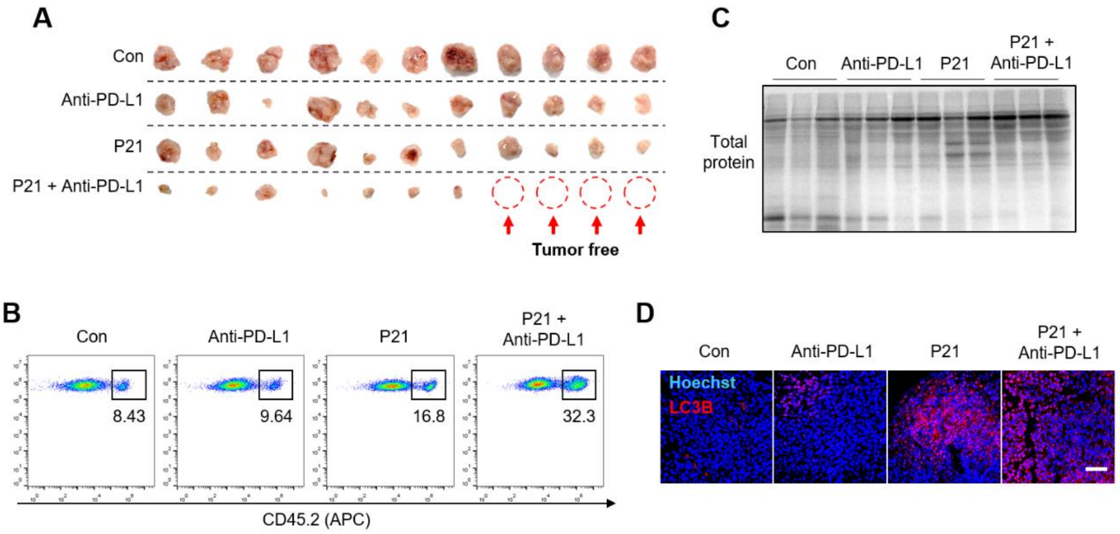

Figure S4. (A) Representative photographs of tumors after 22 days of treatment (*n* = 11/group). (B) Representative flow cytometry analysis of the total immune cell ($CD45.2^{+}$) proportion in the TME. (C) Coomassie staining of the total protein abundance to normalize HMGB1 expression in tumor tissues (*n* = 3/group). (D) Representative immunofluorescence images of LC3B expression (red) in tumor tissues. The nuclei were stained with Hoechst 33342 (blue) (scale bar = 50 µm; *n* = 3-4/group).
